# Phosphorylation-related genes in lupus nephritis: Single-cell and machine learning insights

**DOI:** 10.1016/j.gendis.2024.101385

**Published:** 2024-08-06

**Authors:** Lisha Mou, Zhihao Chen, Xinran Tian, Yupeng Lai, Zuhui Pu, Meiying Wang

**Affiliations:** a Department of Rheumatology and Immunology, Institute of Translational Medicine, Health Science Center, The First Affiliated Hospital of Shenzhen University, Shenzhen Second People's Hospital, Shenzhen, Guangdong 518035, China; b MetaLife Center, Shenzhen Institute of Translational Medicine, Shenzhen, Guangdong 518035, China; c Imaging Department, The First Affiliated Hospital of Shenzhen University, Shenzhen Second People's Hospital, Shenzhen, Guangdong 518035, China

This study investigates key genes contributing to lupus nephritis (LN). While extensive research has elucidated various aspects of LN pathogenesis, the specific involvement of phosphorylation-related genes (PRGs) in this context remains an area of growing interest. We employ single-cell RNA sequencing analysis on renal tissues from 24 LN patients and 10 healthy controls. Leveraging the non-negative matrix factorization (NMF) algorithm, we identified critical gene patterns and constructed 61 predictive models using a comprehensive suite of 12 machine learning algorithms. We developed a predictive model using 6 PRGs, enhanced by a LASSO plus Naive Bayes approach. These PRGs demonstrated high predictive accuracy (the area under a receiver operating characteristic curve was 0.954 and 0.845 in training and validation cohorts, respectively). One of these PRGs, *CEBPB* (CCAAT enhancer binding protein beta), showed significant differences in expression between LN patients and controls. Further analysis revealed correlations between *CEBPB* and renal function parameters. The insights of this study into gene expression patterns enhance our understanding of the molecular mechanisms and support the potential for early diagnosis and targeted treatment strategies in LN. Extensive protein–protein interaction network analysis and clinical correlations underscore the important roles of these genes in LN. Our findings underscore the potential of PRGs as biomarkers, propelling the precision medicine frontier in LN diagnostics and therapeutics.

The intricate interplay of signaling pathways and molecular events, including phosphorylation processes mediated by PRGs, holds key implications for understanding the molecular underpinnings of LN. Recent advancements in single-cell RNA sequencing have significantly enhanced our ability to analyze cellular and gene expression diversity in detail. Our study utilizes NMF^1^^,^^2^ and machine learning algorithms to transform complex biological data into clear patterns of LN pathology, focusing on PRGs as potential biomarkers or therapeutic targets. At the intersection of high-throughput sequencing and bioinformatics, this approach advances precision medicine in LN, promoting personalized treatment strategies based on unique molecular profiles.

The study began with the analysis of single-cell data derived from 24 LN patients and 10 healthy control participants accessed from the previous study.^3^ These analyses led to the identification of distinct cellular clusters representing LN and control cohorts, with a focus on five major cell types (Fig. 1A) and 22 sub-cell types (Fig. 1B). Five major cell types include T cells, natural killer cells, myeloid cells, B cells, and epithelial cells (Fig. 1A; Fig. S1A). The relative cell proportions in the LN and control group are presented in Figure S1B and in each patient are shown in Figure S1C. Importantly, differential gene expression analysis revealed key genes with significant expression differences between LN and healthy control groups (Fig. S2A).

**Figure 1:**
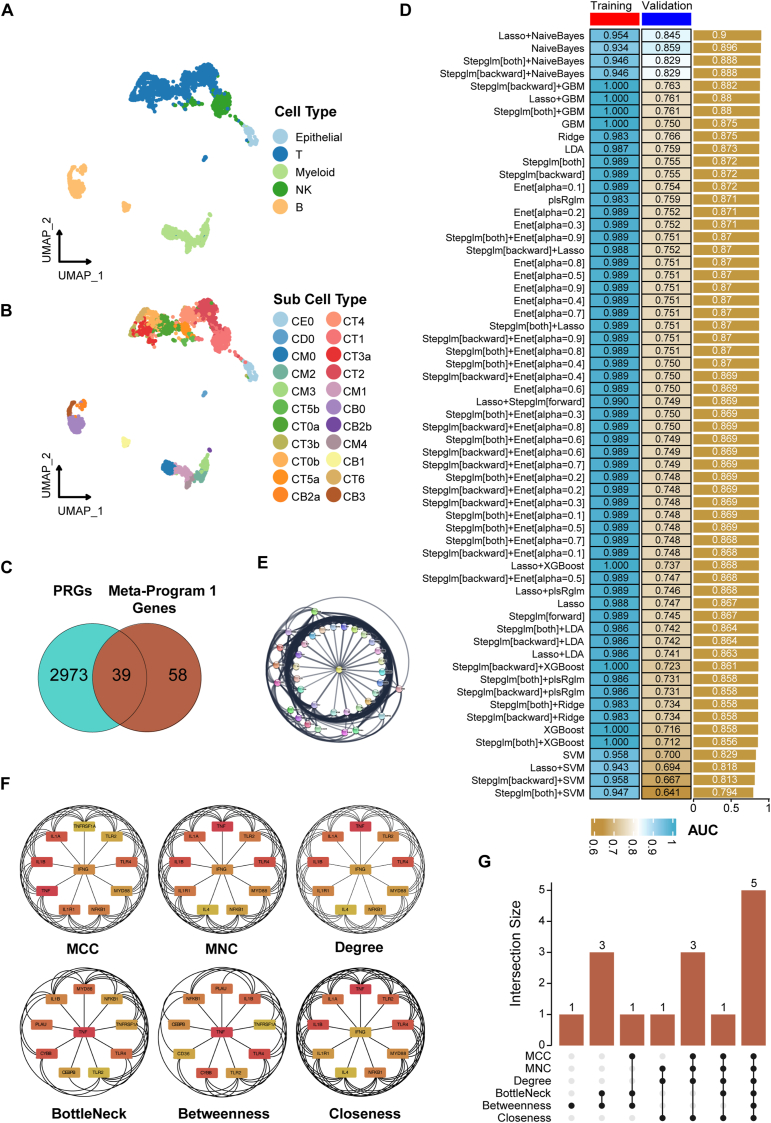
Development of a phosphorylation-related predictive model in lupus nephritis (LN). **(A)** The UMAP plot of five cell types of renal tissues from 24 LN patients and 10 healthy controls in the single-cell dataset. **(B)** The UMAP plot detailing 22 sub-cell types. **(C)** The Venn diagram highlighting 39 intersecting phosphorylation-related genes (PRGs) with Meta-Program 1 genes. **(D)** The results of the area under a receiver operating characteristic curve (AUC) for 61 algorithm combinations (the training cohort was from GSE32591 and GSE113342 datasets, and the validation cohort was from GSE200306). **(E)** Protein–protein interaction network analysis. A network diagram based on the 30 most interconnected genes with six hub PRGs. **(F)** Identification of the top ten hub genes using six analytical algorithms, including Maximum Clique Centrality (MCC), Maximum Neighborhood Component (MNC), Degree, BottleNeck, Betweenness, and Closeness. **(G)** The UpSet plot showed five overlapping genes identified by all algorithms.

The transcriptional landscape of leukocytes in LN samples was uncovered using the NMF algorithm^4^ to identify four distinct meta-programs. These meta-programs, referred to as Meta-Program 1, Meta-Program 2, Meta-Program 3, and Meta-Program 4, were characterized by sets of top-scoring genes. Meta-Program 1, for example, included *CD14* (cluster of differentiation 14), *CYBB* (cytochrome B-245 beta chain), and *CD36* (cluster of differentiation 36) as key genes. A comprehensive analysis, including gene module identification and hierarchical clustering, facilitated the categorization of leukocyte expression programs (Fig. S2B). These findings showcased the genetic heterogeneity within LN leukocytes and offered insights into the cellular landscape.

To develop an accurate predictive PRG model for LN, 39 PRGs were extracted from the intersection of Meta-Program 1 and PRGs, which were acquired from the Gene Ontology database (Fig. 1C). These 39 PRGs exhibited notable expression patterns, with a strong representation in myeloid cells (Fig. S2C). Utilizing twelve machine-learning algorithms,^5^ including LASSO, Ridge, Elastic network, Stepglm, SVM, GlmBoost, LDA, plsRglm, RSF, GBMs, XGBoost, and Naive Bayes, we constructed 61 PRG predictive models (Fig. 1D). These models encompassed various gene sets and configurations. The top-performing LASSO plus Naive Bayes model demonstrated exceptional performance incorporating six genes, including *CD14*, *CD36*, *CEBPB*, *CYBB*, *IL1B* (interleukin 1 beta), and *PLAUR* (plasminogen activator, urokinase receptor), achieving values of 0.954 and 0.845 for the area under a receiver operating characteristic curve in the training and validation renal cohorts, respectively. The expression of these six hub PRGs in single-cell datasets is shown in Figure S3. The results showed that all these six hub genes are highly expressed in myeloid cells, while *CYBB* was also highly expressed in B cells (Fig. S3).

We conducted a protein–protein interaction network analysis of the six hub PRGs to reveal their functional relationships. Using the STRING database, we built a robust protein–protein interaction network focusing on the top 30 genes connected to the hub PRGs with high–confidence interactions (confidence scores >0.7) (Fig. 1E). Data were imported into Cytoscape and analyzed with cytoHubba plugins, identifying the top ten nodes using six algorithms (Maximum Clique Centrality, Maximum Neighborhood Component, Degree, BottleNeck, Betweenness, Closeness) (Fig. 1F). This multi-algorithm approach provided a comprehensive analysis of the network's key components.

Our UpSet diagram revealed five common hub genes: *TNF* (tumor necrosis factor), *IL1B*, *TLR4* (Toll-like receptor 4), *TLR2* (Toll-like receptor 2), and *NFKB1* (nuclear factor kappa B subunit 1), indicating their significant roles in the regulatory network of LN (Fig. 1G). The convergence of these genes highlights their potential collaborative role in disease pathogenesis, warranting further exploration. This rigorous analysis enhances our understanding of the functional roles and interactions of the selected PRGs.

In our comprehensive investigation of LN, we rigorously analyzed the expression levels of *CEBPB* using a robust dataset of 63 samples sourced from the Ju Tubulointerstitium cohort. Our validation process revealed a notable down-regulation of *CEBPB* in LN patients when compared with healthy controls, as depicted in Figure S4A. In this context, the differential expression of *CEBPB* is a critical discovery, highlighting the gene's significant modulation in the disease state.

Delving deeper into the clinical ramifications of *CEBPB* expression, we performed a detailed correlation analysis to examine its relationship with key renal function parameters using the ERCB Tubulointerstitium cohort. This analysis was not only pivotal in establishing a link between *CEBPB* expression levels and disease pathology but also in underscoring the gene's regulatory impact on renal function. Remarkably, our data elucidated a consistent negative correlation between *CEBPB* expression and glomerular filtration rate, a primary indicator of renal health, as illustrated in Figure S4B.

*CEBPB* regulates immune responses and inflammation. In LN patients, its down-regulation correlates with decreased renal function and lower glomerular filtration rate, suggesting a protective role and potential as a biomarker for LN severity.

The other hub PRGs—*CD14*, *CD36*, *CYBB*, *IL1B*, and *PLAUR*—are involved in metabolic, inflammatory, and immune pathways in LN. Increased *CD14* may exacerbate inflammation and tissue damage. *CD36* is linked to renal cell damage through metabolic disturbances. Elevated *CYBB* indicates oxidative stress. *IL1B* contributes to kidney inflammation, and *PLAUR* is involved in chronic inflammation and tissue damage.

This study has limitations. Functional validations are needed to confirm the pathways identified. *In vitro* and *in vivo* studies are required to assess the roles of key PRGs using techniques like CRISPR-Cas9 and proteomics. Further research will help establish causal relationships between gene expression changes and disease phenotypes.

These findings highlight the complex interplay between metabolic, inflammatory, and immune pathways in LN. This study provides a foundation for future research on targeted therapies, offering robust diagnostic models and potential therapeutic targets. The integration of single-cell data, machine learning, and protein–protein interaction analyses contributes to a holistic understanding of LN and informs improved clinical interventions and patient outcomes.

## Funding

This work was supported by the Science and Technology Program for Basic Research in Shenzhen, Guangdong, China (No. JCYJ20200109140412476, JCYJ20190809095811254, GCZX2015043017281705), the Clinical Research Project in Shenzhen, Guangdong, China (No. 20213357002, 20213357028), and the Team-based Medical Science Research Program in Shenzhen, Guangdong, China (No. 2024YZZ06), and Shenzhen High-level Hospital Construction Fund in Shenzhen, Guangdong, China (No. 2024).

## Author contributions

**Lisha Mou:** Conceptualization, Formal analysis, Writing – original draft, Writing – review & editing, Funding acquisition. **Zhihao Chen:** Formal analysis. **Xinran Tian:** Formal analysis. **Yupeng Lai:** Writing – review & editing, Funding acquisition. **Zuhui Pu:** Conceptualization, Writing – review & editing. **Meiying Wang:** Writing – review & editing, Funding acquisition.

## Conflict of interests

The authors declared no competing interests.

## Figures and Tables

**figs1:**
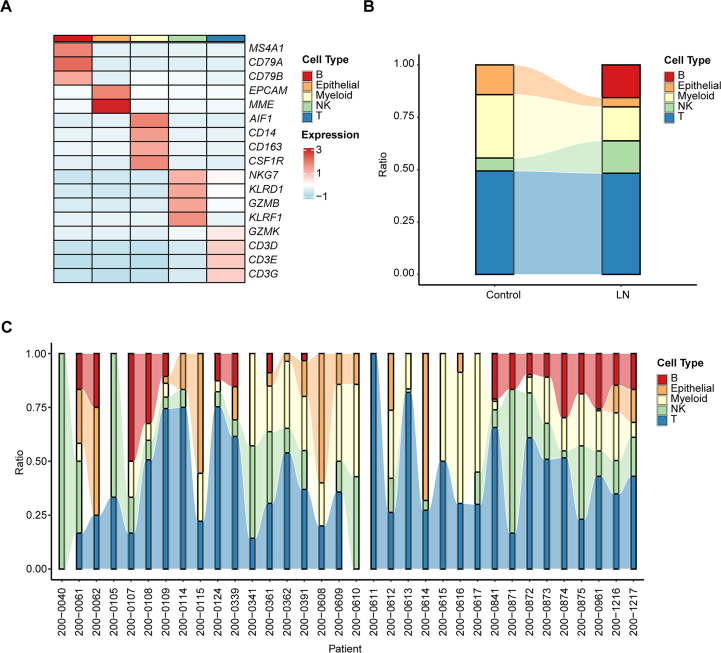
Figure S1. Single-cell RNA sequencing analysis in lupus nephritis (LN).

**figs2:**
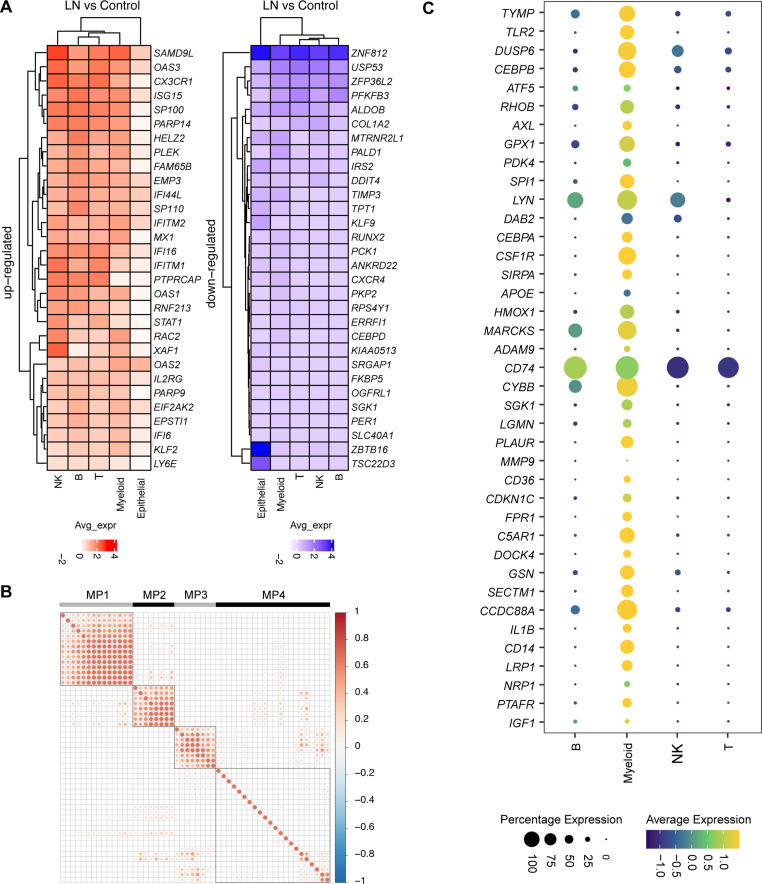
Figure S2. Meta-Programs and phosphorylation-related genes (PRGs).

**figs3:**
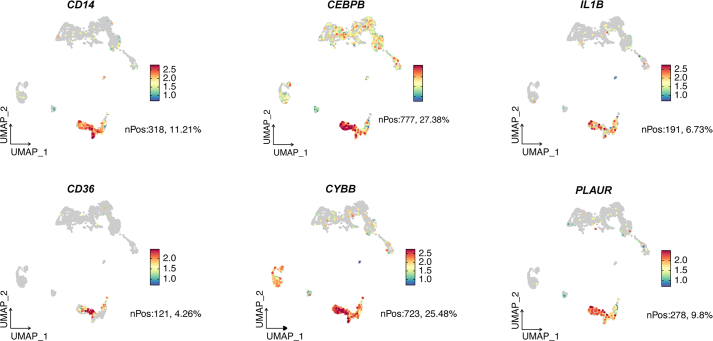
Figure S3. The UMAP plot of six hub PRG expressions across different cells in single-cell data.

**figs4:**
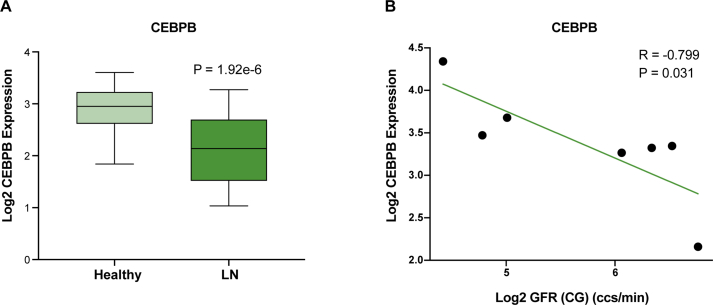
Figure S4. Validation of CEBPB and clinical correlation with renal function.
