# Supplementary material for: Phosphorylation-related genes in lupus nephritis: Single-cell and machine learning insights
Source: Genes Dis. 2024 Aug 6;12(3):101385. doi: 10.1016/j.gendis.2024.101385 (PMC11786865; doi:10.1016/j.gendis.2024.101385)
Supplement: Multimedia component 1 [file mmc1.docx]

**1. Important Materials and Methods**

**1.1 Data Processing**

Single-cell RNA sequencing (scRNA-seq) data from twenty-four lupus nephritis (LN) patients and ten control samples were obtained from the previous study (1). Additionally, bulk RNA datasets were retrieved from the Gene Expression Omnibus (GEO) database, comprising three cohorts of LN patients: GSE32591 (64 LN samples and 29 healthy renal samples, based on the GPL14663 platform) (2), GSE113342 (56 LN samples and 16 healthy renal samples, based on the GPL21847 platform) (3), GSE200306 (79 LN samples with first biopsy and 19 healthy renal samples, based on the GPL21847 platform) (4), Phosphorylation-related genes (PRGs) were acquired from the Gene Ontology database.

- 1. **Single-Cell Data Analysis**

The investigation commenced with an examination of single-cell data extracted from 24 LN patients and 10 healthy controls. Employing Seurat (5) for filtering and clustering, the analysis enabled the identification of discrete cellular clusters representing LN and control cohorts. We refer to the original article for cell quality control and annotation. This comprehensive analysis focused on characterizing five major cell types (T cells, NK cells, myeloid cells, B cells, epithelial cells) and 22 sub-cell types. Furthermore, the differential gene expression analysis uncovered key genes exhibiting significant expression disparities between the LN and healthy control groups. This approach, utilizing the Wilcoxon method with adjusted P-values set at 0.05 and an absolute log2FC value exceeding 1, provided crucial insights into the genetic alterations underlying Lupus Nephritis.

**1.3 Non-Negative Matrix Factorization (NMF) and Meta-Program Detection of Leukocytes in LN Patients**

For the analysis of leukocytes in LN samples, the consensus Non-negative Matrix Factorization (cNMF) algorithm was utilized. Negative values were standardized to zero. The NMF analysis was executed independently for leukocytes in each LN sample. The optimal number of components (k) was determined using the diagnostic plot approach from the provided tutorial (<https://github.com/dylkot/cNMF>) (6). To identify non-overlapping gene modules, we used a gene ranking algorithm, involving the construction of two ranking matrices. The first-ranked gene contributes to each factor (list 1), and the second-ranked factors contribute to each gene (list 2). Genes were incrementally added for each factor following their contribution (list 1) until reaching a point where their contribution to another factor superseded, as indicated by their rank across factors (list 2) not being 1. Expression program patterns were further analyzed by employing Pearson correlations and conducting hierarchical clustering, resulting in the identification of four distinct meta-programs.

**1.4 Development of the PRGs Predictive Model for LN**

In pursuit of a highly accurate and generalizable predictive model for LN, we focused on the intersection of Meta-Program 1 and PRGs. To construct robust predictive models, we employed a diverse set of twelve machine-learning algorithms, including LASSO, Ridge, Elastic network, Stepglm, SVM, GlmBoost, LDA, plsRglm, RSF, GBMs, XGBoost, and Naive Bayes. Predictive models constructed by GlmBoost and RSF with lower performance metrics were excluded. Finally, a total of 61 PRG predictive models were created, each representing various gene sets and configurations. Efficiency was a key focus in model development, with the chosen models emphasizing diagnostic accuracy while focusing on key genes. The ROC curve analysis illustrated the predictive performance of hub PRGs. In the initial phase of model construction, we strategically utilized a training dataset comprising a combination of the GSE32591 and GSE113342 cohorts. This merged dataset facilitated the preliminary development of predictive models, capitalizing on the diversity and richness of information encapsulated within these cohorts. Following the construction of predictive models, the next crucial step involved their validation in an independent cohort of GSE200306. This validation process was instrumental in evaluating the robustness and generalizability of the models across distinct datasets, ensuring that the predictive power observed in the training phase could be effectively extended to diverse samples. The performance assessment of the constructed models was quantified using the Area Under The Curve (AUC), a widely acknowledged metric for evaluating predictive accuracy. By calculating the AUC for each model across all cohorts, we obtained a comprehensive understanding of their discriminative ability and overall performance in different datasets. This rigorous evaluation process further ensured the reliability and applicability of the predictive models, laying the groundwork for their potential integration into clinical practice.

**1.5 Protein Interaction Network Analysis of Hub PRGs**

In this study, we conducted an in-depth exploration of protein-protein interactions involving six crucial PRGs, aiming to unravel their functional relationships and cellular significance. Leveraging the STRING database (https://string-db.org/), we systematically compiled information on protein interactions, focusing on interactions with confidence scores exceeding 0.7 for robustness and significance in our analysis. To facilitate visualization and gain insights into the complex network, we seamlessly imported the data into Cytoscape (version 3.8.2). Within Cytoscape, we harnessed the power of cytoHubba plugins to identify the top 10 nodes within the Protein-Protein Interaction (PPI) network. These nodes were ranked using six distinct algorithms, including Maximum Clique Centrality (MCC), Maximum Neighborhood Component (MNC), Degree, BottleNeck, Betweenness, and Closeness. The integration of these diverse algorithms ensured a comprehensive analysis of the most important components in the network. The convergence of hub genes identified by different algorithms was visually represented using the UpSet diagram, offering a clear and concise overview of the overlapping hub genes.

**1.6 Validation and Clinical Correlation Analysis of *CEBPB***

To validate the expression profiles of *CEBPB*, we utilized a comprehensive dataset comprising 63 samples from the Ju Tubulointerstitium cohort (7). Expanding our analysis to explore the clinical correlations of *CEBPB* with essential renal function parameters within LN patients, we sourced information from the ERCB Tubulointerstitium cohort (8). Utilizing datasets from the ERCB Lupus Tubulointerstitium RNA-Seq, which included a comprehensive sample set of 41 cases, we analyzed the correlation between *CEBPB* expression and glomerular filtration rate (GFR).

## 1.7 Statistical Analysis

Statistical analyses for both single-cell and bulk RNA-seq data were conducted using R (Version 4.3.1). The significance threshold for all analyses was set at a P-value less than 0.05, indicating statistical significance.

**2 Additional Discussion**

The comprehensive exploration of Phosphorylation-Related Genes (PRGs) in Lupus Nephritis (LN) has provided valuable insights into the molecular landscape of this complex autoimmune renal disorder. The findings presented in this study contribute to our understanding of the role of PRGs in LN pathogenesis and open avenues for further research and clinical applications. The integration of single-cell RNA sequencing data has allowed for the identification of key PRGs associated with LN. The development of predictive models, particularly the LASSO plus NaiveBayes model, showcases the potential of PRGs as diagnostic biomarkers for LN. The robust performance of this model, incorporating *CD14*, *CD36*, *CEBPB*, *CYBB*, *IL1B*, and *PLAUR*, underscores the specificity and sensitivity of these PRGs in distinguishing LN patients from healthy controls.

The interplay between *CD14* and LN is significant due to *CD14*'s critical role in innate immunity and its association with systemic lupus erythematosus (SLE). The *CD14* (C-159T) polymorphism is linked to increased SLE risk, highlighting its potential impact on autoimmune diseases (9). Altered expression of Fas and Bcl-2 proteins in *CD14*+ monocytes in juvenile SLE suggests dysregulation in *CD14*-related pathways may influence LN pathophysiology. Increased expression of *CD14* in LN patients could heighten immune activity, exacerbating inflammation and tissue damage (10).

*CD36*, known for its role in lipid metabolism and inflammation, exacerbates podocyte damage through NLRP3 inflammasome activation and autophagy inhibition (11). Its role in foam cell formation via the p38 MAPK pathway links *CD36* to lipid metabolism disturbances, contributing to LN pathology (12). *CD36* has been identified as a hub gene in LN, underscoring its significance in disease pathogenesis (13).

*CEBPB* regulates immune responses and inflammation (14). Our analysis revealed down-regulation of *CEBPB* in LN patients, correlating with decreased renal function. This suggests a protective role for *CEBPB*, with its reduced expression contributing to renal pathology progression in LN. Detailed correlation analysis showed a consistent negative correlation between *CEBPB* expression and glomerular filtration rate (GFR), indicating its potential as a biomarker for LN severity and progression.

*CYBB*, involved in the production of reactive oxygen species (ROS) during the respiratory burst of phagocytes, shows elevated expression in LN patients, pointing to increased oxidative stress that can damage renal tissues. Contradictory findings regarding CYBB and neutrophil extracellular traps (NETs) indicate a more nuanced role in lupus than previously thought (15).

*IL1B* is a pro-inflammatory cytokine upregulated in LN patients, contributing to kidney inflammation (16). Its involvement in inflammatory pathways makes it a potential therapeutic target.

*PLAUR*, linked to tissue remodeling and immune regulation, is elevated in LN patients, suggesting its role in chronic inflammation and tissue damage (17). *PLAUR's* involvement in kidney pathology reinforces its relevance in LN (18).

*CEBPB* is associated with the regulation of immune responses and inflammation. Our analysis revealed a down-regulation of *CEBPB* in LN patients, which correlates with decreased renal function. This suggests that *CEBPB* may have a protective role, and its reduced expression could contribute to the progression of renal pathology in LN. Detailed correlation analysis showed a consistent negative correlation between *CEBPB* expression and GFR. This inverse relationship indicates that lower *CEBPB* levels are associated with impaired kidney function, suggesting its potential as a biomarker for disease severity and progression in LN patients.

The remaining hub PRGs including *CD14*, *CD36*, *CYBB*, *IL1B*, and *PLAUR* are also involved in metabolic, inflammatory, and immune pathways in LN. *CD14* is a crucial component of the innate immune system, playing a significant role in recognizing pathogens and initiating immune responses. Our findings suggest that the increased expression of *CD14* in LN patients could contribute to the heightened immune activity observed in LN, potentially exacerbating inflammation and tissue damage. *CD36* is known for its role in lipid metabolism and inflammation. It is implicated in podocyte injury through the activation of the NLRP3 inflammasome. Our data indicate that *CD36* may serve as a mediator of renal cell damage in LN, linking metabolic disturbances with inflammatory pathways. *CYBB* is involved in the production of reactive oxygen species (ROS) during the respiratory burst of phagocytes. Its elevated expression in LN patients points to increased oxidative stress, which can damage renal tissues and exacerbate LN symptoms. *IL1B* is a pro-inflammatory cytokine that is upregulated in LN patients, contributing to the inflammatory milieu of the kidney. Its involvement in inflammatory pathways makes it a potential target for therapeutic intervention. *PLAUR* is linked to tissue remodeling and immune regulation. Elevated levels of *PLAUR* in LN patients suggest its role in the chronic inflammation and tissue damage characteristic of LN.

This study has several limitations. Functional validations are needed to confirm the candidate pathways identified. In vitro and in vivo studies are required to assess the roles of key PRGs such as *CEBPB*, *CD36*, and *CYBB* in LN, using techniques like CRISPR-Cas9, overexpression, and knockdown experiments. Further investigation into protein-protein interactions and downstream signaling effects through proteomics and pathway analysis is necessary to establish causal relationships between gene expression changes and disease phenotypes, enhancing the translational potential of our findings.

The convergence of these findings underscores the complex interplay between metabolic, inflammatory, and immune pathways in LN. By elucidating the roles of these PRGs, our study provides a foundation for future research aimed at developing targeted therapies. In summary, this study offers a comprehensive understanding of the molecular landscape of lupus nephritis, presenting robust diagnostic models, potential therapeutic targets, and valuable insights into the underlying pathophysiology. The integration of single-cell data, meta-program identification, machine learning, and protein interaction analyses contributes to a holistic perspective on LN and sheds light on improved clinical interventions and patient outcomes.

The validation of PRG expression patterns in larger datasets, such as the Ju Tubulointerstitium cohort, reaffirms the clinical relevance of *CEBPB* in LN. The observed correlations with renal function parameters, including glomerular filtration rate (GFR), further highlight the potential functional significance of PRGs in disease progression. The negative correlation of *CEBPB* with GFR with renal parameters underscores the roles of *CEBPB* in renal dysfunction. The identification of *CD14*, *CD36*, *CEBPB*, *CYBB*, *IL1B*, and *PLAUR* as hub PRGs in LN represents a significant advancement in our understanding of the molecular underpinnings of this complex autoimmune renal disorder. The differential expression and clinical correlations of these hub PRGs in LN patients, as validated in larger datasets, highlight their potential as clinically relevant biomarkers. The functional characterization of these hub PRGs and their integration into the broader context of LN pathogenesis opens avenues for precision medicine approaches, emphasizing the need for the advancement of diagnostic strategies with improved accuracy.

The Protein-Protein Interaction (PPI) network analysis has unveiled a complex interplay among the identified PRGs. The convergence of hub genes, such as *TNF*, *IL1B*, *TLR4*, *TLR2*, and *NFKB1*, emphasizes their interconnectedness and potential collaborative roles in LN pathogenesis. *TNF*, a key pro-inflammatory cytokine, regulates immune cell function, apoptosis, and inflammation (19). Its dysregulation is crucial in the pathogenesis of LN pathogenesis, with anti-*TNF* therapies proving beneficial in some LN cases, underlining the therapeutic relevance of *TNF* (20). The role of *TLR4* as an innate immunity receptor and its contribution to LN development necessitates further investigation to elucidate its complex relationship with SLE (21,22). Similarly, *TLR2*, another innate immunity receptor recognizing microbial patterns, is involved in immune activation and inflammatory responses, with variations in *TLR2* expression and polymorphisms linked to SLE, suggesting its participation in the aberrant immune responses of LN (23–25). *NFKB1*, a transcription factor, is increasingly recognized in LN pathology, with its dysregulation correlating with the disease and genetic variations influencing SLE susceptibility (26). *NFKB1* is a candidate for potential therapeutic intervention and as a biomarker for this complex condition. Collectively, these genes form a network that contributes to the immune dysregulation of LN, offering a window into the disease mechanism and informing the development of targeted treatments and diagnostics.

It is essential to acknowledge the limitations of this study. Further investigations should focus on unraveling the dynamic changes in the expression of these PRGs during the course of LN, considering longitudinal studies to capture the temporal evolution of their involvement. Additionally, further validation in diverse cohorts and functional experiments is warranted to elucidate the precise mechanisms through which these PRGs contribute to LN pathology.

It is crucial to acknowledge the complexity of LN pathogenesis and the multifactorial nature of autoimmune disorders. While the identified hub PRGs shed light on specific molecular players, LN is likely influenced by a network of interconnected factors. Integration with other 'omics' data, such as proteomics and metabolomics, may offer a more comprehensive understanding of the molecular landscape in LN.

In conclusion, the elucidation of PRGs in LN through a multi-faceted approach has laid the foundation for a more nuanced understanding of the molecular mechanisms driving this autoimmune renal disorder. The identified PRGs not only serve as potential diagnostic markers but also offer insights into the intricate interplay of signaling pathways in LN. This study sets the stage for future investigations aimed at translating these molecular insights into targeted therapies, ultimately advancing the prospects for precision medicine in LN.

**Aberration**

Systemic Lupus Erythematosus (SLE)

Lupus Nephritis (LN)

Phosphorylation-related genes (PRGs)

Non-negative Matrix Factorization (NMF)

Single-cell RNA sequencing (scRNA-seq)

Area Under The Curve (AUC)

Glomerular filtration rate (GFR)

Protein-Protein Interaction (PPI)

Maximum Clique Centrality (MCC)

Maximum Neighborhood Component (MNC)

**Reference**

1. Arazi A, Rao DA, Berthier CC, Davidson A, Liu Y, Hoover PJ, Chicoine A, Eisenhaure TM, Jonsson AH, Li S, et al. The immune cell landscape in kidneys of patients with lupus nephritis. *Nat Immunol* (2019) 20:902–914. doi: 10.1038/s41590-019-0398-x

2. Berthier CC, Bethunaickan R, Gonzalez-Rivera T, Nair V, Ramanujam M, Zhang W, Bottinger EP, Segerer S, Lindenmeyer M, Cohen CD, et al. Cross-species transcriptional network analysis defines shared inflammatory responses in murine and human lupus nephritis. *J Immunol* (2012) 189:988–1001. doi: 10.4049/jimmunol.1103031

3. Mejia-Vilet JM, Parikh SV, Song H, Fadda P, Shapiro JP, Ayoub I, Yu L, Zhang J, Uribe-Uribe N, Rovin BH. Immune gene expression in kidney biopsies of lupus nephritis patients at diagnosis and at renal flare. *Nephrol Dial Transplant* (2019) 34:1197–1206. doi: 10.1093/ndt/gfy125

4. Zhu H, Mi W, Luo H, Chen T, Liu S, Raman I, Zuo X, Li Q-Z. Whole-genome transcription and DNA methylation analysis of peripheral blood mononuclear cells identified aberrant gene regulation pathways in systemic lupus erythematosus. *Arthritis Res Ther* (2016) 18:162. doi: 10.1186/s13075-016-1050-x

5. Hao Y, Hao S, Andersen-Nissen E, Mauck WM, Zheng S, Butler A, Lee MJ, Wilk AJ, Darby C, Zager M, et al. Integrated analysis of multimodal single-cell data. *Cell* (2021) 184:3573-3587.e29. doi: 10.1016/j.cell.2021.04.048

6. Kotliar D, Veres A, Nagy MA, Tabrizi S, Hodis E, Melton DA, Sabeti PC. Identifying gene expression programs of cell-type identity and cellular activity with single-cell RNA-Seq. *Elife* (2019) 8:e43803. doi: 10.7554/eLife.43803

7. Ju W, Greene CS, Eichinger F, Nair V, Hodgin JB, Bitzer M, Lee Y-S, Zhu Q, Kehata M, Li M, et al. Defining cell-type specificity at the transcriptional level in human disease. *Genome Res* (2013) 23:1862–1873. doi: 10.1101/gr.155697.113

8. Grayson PC, Eddy S, Taroni JN, Lightfoot YL, Mariani L, Parikh H, Lindenmeyer MT, Ju W, Greene CS, Godfrey B, et al. Metabolic pathways and immunometabolism in rare kidney diseases. *Ann Rheum Dis* (2018) 77:1226–1233. doi: 10.1136/annrheumdis-2017-212935

9. Panda AK, Tripathy R, Das BK. CD14 (C-159T) polymorphism is associated with increased susceptibility to SLE, and plasma levels of soluble CD14 is a novel biomarker of disease activity: a hospital-based case-control study. *Lupus* (2021) 30:219–227. doi: 10.1177/0961203320972799

10. Liphaus B, Kiss M, Carrasco S, Goldenstein-Schainberg C. Reduced expressions of Fas and Bcl-2 proteins in CD14+ monocytes and normal CD14 soluble levels in juvenile systemic lupus erythematosus. *Lupus* (2013) 22:940–947. doi: 10.1177/0961203313496300

11. Lv F, He Y, Xu H, Li Y, Han L, Yan L, Lang H, Zhao Y, Zhao Z, Qi Y. CD36 aggravates podocyte injury by activating NLRP3 inflammasome and inhibiting autophagy in lupus nephritis. *Cell Death Dis* (2022) 13:729. doi: 10.1038/s41419-022-05179-9

12. Liu Q, Fan J, Bai J, Peng L, Zhang T, Deng L, Wang G, Zhao Y, Jingguo N, Zhang M, et al. IL-34 promotes foam cell formation by enhancing CD36 expression through P38 MAPK pathway. *Scientific Reports* (2018) doi: 10.1038/s41598-018-35485-2

13. Yang H, Li H. CD36 identified by weighted gene co-expression network analysis as a hub candidate gene in lupus nephritis. *Peerj* (2019) doi: 10.7717/peerj.7722

14. Wang X, Cheng W, Chen X, Gong Y, Wang G, Zhang X, Qi Y. Inhibition of CEBPB attenuates lupus nephritis via regulating Pim-1 signaling. *Mediators of Inflammation* (2022) 2022:1–14. doi: 10.1155/2022/2298865

15. Gordon RA, Herter JM, Rosetti F, Campbell AM, Nishi H, Kashgarian M, Bastacky SI, Marinov A, Nickerson KM, Mayadas TN, et al. Lupus and proliferative nephritis are PAD4 independent in murine models. *JCI Insight* (2017) 2:e92926. doi: 10.1172/jci.insight.92926

16. Tang Y, Zhang Y, Li X, Xu R, Ji Y, Liu J, Liu J, Zhuang Q, Zhang H. Immune landscape and the key role of APOE+ monocytes of lupus nephritis under the single‐cell and spatial transcriptional vista. *Clin Transl Med* (2023) 13:e1237. doi: 10.1002/ctm2.1237

17. Dowsett J, Ferkingstad E, Rasmussen LJH, Thørner LW, Magnússon MK, Sugden K, Thorleifsson G, Frigge M, Burgdorf KS, Ostrowski SR, et al. Eleven genomic loci affect plasma levels of chronic inflammation marker soluble urokinase-type plasminogen activator receptor. *Commun Biol* (2021) 4:655. doi: 10.1038/s42003-021-02144-8

18. Narayanaswamy PB, Baral TK, Haller H, Dumler I, Acharya K, Kiyan Y. Transcriptomic pathway analysis of urokinase receptor silenced breast cancer cells: a microarray study. *Oncotarget* (2017) 8:101572–101590. doi: 10.18632/oncotarget.21351

19. Jang D, Lee A-H, Shin H-Y, Song H-R, Park J-H, Kang T-B, Lee S-R, Yang S-H. The role of tumor necrosis factor alpha (TNF-α) in autoimmune disease and current TNF-α inhibitors in therapeutics. *IJMS* (2021) 22:2719. doi: 10.3390/ijms22052719

20. Olaru F, Döbel T, Lonsdorf AS, Oehrl S, Maas M, Enk AH, Schmitz M, Gröne EF, Gröne H-J, Schäkel K. Intracapillary immune complexes recruit and activate slan-expressing CD16+ monocytes in human lupus nephritis. *JCI Insight* (2018) 3:e96492. doi: 10.1172/jci.insight.96492

21. Ma K, Li J, Wang X, Lin X, Du W, Yang X, Mou F, Fang Y, Zhao Y, Hong X, et al. TLR4 + CXCR4 + plasma cells drive nephritis development in systemic lupus erythematosus. *Ann Rheum Dis* (2018) 77:1498–1506. doi: 10.1136/annrheumdis-2018-213615

22. Rekvig OP, Thiyagarajan D, Pedersen HL, Horvei KD, Seredkina N. Future perspectives on pathogenesis of lupus nephritis. *The American Journal of Pathology* (2016) 186:2772–2782. doi: 10.1016/j.ajpath.2016.06.026

23. Elloumi N, Tahri S, Fakhfakh R, Abida O, Mahfoudh N, Hachicha H, Marzouk S, Bahloul Z, Masmoudi H. Role of innate immune receptors TLR4 and TLR2 polymorphisms in systemic lupus erythematosus susceptibility. *Annals of Human Genetics* (2022) 86:137–144. doi: 10.1111/ahg.12458

24. Gao Y, Xiao H, Wang Y, Xu F. Association of single-nucleotide polymorphisms in toll-like receptor 2 gene with asthma susceptibility: A meta-analysis. *Medicine* (2017) 96:e6822. doi: 10.1097/MD.0000000000006822

25. Wu Y, Tang W, Zuo J. Toll-like receptors: potential targets for lupus treatment. *Acta Pharmacol Sin* (2015) 36:1395–1407. doi: 10.1038/aps.2015.91

26. Zou YF, Wang F, Feng XL, Tao JH, Zhu JM, Pan FM, Su H. Association of NFKB1 ‐94ins/delATTG promoter polymorphism with susceptibility to autoimmune and inflammatory diseases: a meta‐analysis. *Tissue Antigens* (2011) 77:9–17. doi: 10.1111/j.1399-0039.2010.01559.x

**Supplementary Figure Legends**

**Figure S1**. Single-cell RNA sequencing analysis in lupus nephritis (LN). **(A)** Heatmap of marker gene expression in five major cell types. **(B)** Comparative stacked bar chart of cell type proportions in LN versus controls. **(C)** Participant-specific stacked bar chart of cell-type distribution.

**Figure S2.** Meta-Programs and phosphorylation-related genes (PRGs) in LN. **(A)** Heatmap contrasting gene expression in LN versus controls. **(B)** Identification of four meta-programs: meta-program 1 (MP1), meta-program 2 (MP2), meta-program 3 (MP3), and meta-program 4 (MP4). **(C)** Dotplot depicting the expression of 39 PRGs across immune cells in single-cell data.

**Figure S3.** The UMAP plot of six hubs PRG expressions across different cells in single-cell data.

**Figure S4.** Validation of *CEBPB* and clinical correlation with renal function. **(A)** Box plots showing expression levels of *CEBPB* in LN patients versus controls in external cohorts of Ju Tubulointerstitium cohort. **(B)** Scatter plots illustrating correlations of *CEBPB* with glomerular filtration rate (GFR) in external cohorts of ERCB Tubulointerstitium cohort.
